# A Quick Guide for Developing Effective Bioinformatics Programming Skills

**DOI:** 10.1371/journal.pcbi.1000589

**Published:** 2009-12-24

**Authors:** Joel T. Dudley, Atul J. Butte

**Affiliations:** 1 Program in Biomedical Informatics, Stanford University School of Medicine, Stanford, California, United States of America; 2 Department of Pediatrics, Stanford University School of Medicine, Stanford, California, United States of America; 3 Lucile Packard Children's Hospital, Palo Alto, California, United States of America; Whitehead Institute, United States of America

## Introduction

Bioinformatics programming skills are becoming a necessity across many facets of biology and medicine, owed in part to the continuing explosion of biological data aggregation and the complexity and scale of questions now being addressed through modern bioinformatics. Although many are now receiving formal training in bioinformatics through various university degree and certificate programs, this training is often focused strongly on bioinformatics methodology, leaving many important and practical aspects of bioinformatics to self-education and experience. The following set of guidelines distill several key principals of effective bioinformatics programming, which the authors learned through insights gained across many years of combined experience developing popular bioinformatics software applications and database systems in both academic and commercial settings [1]–[6]. Successful adoption of these principals will serve both beginner and experienced bioinformaticians alike in career development and pursuit of professional and scientific goals.

## The Importance of Building Your Technology Toolbox

Given the diversity and complex nature of problems in biology, medicine, and bioinformatics, it is imperative to be able to approach each problem with a comprehensive knowledge of available computational tools—so that the best tools can be selected for the problem at hand. The most fundamental and versatile tools in your technology toolbox are programming languages. While most modern programming languages are capable of any number of computational feats, some are more apt for particular tasks than others. For example, the R language [7] is almost unparalleled in its statistical computing capabilities, whereas the Lisp language is well designed for problems in artificial intelligence, and Erlang [8] excels in fault-tolerant and distributed systems. Given the learning and practice required to become an effective user of a programming language, it is provident to not only gain basic proficiency in a diversity of languages but also to appropriate the time and energy to gain mastery in at least a single language. With programming language mastery comes knowledge and access to advanced language features and libraries, more efficient programming, and less time spent reading manuals and making novice errors.

While there are many languages that would be appropriate and effective in which to seek mastery for bioinformatics, modern interpreted scripting languages, such as Perl [9], Python [10], and Ruby [11], are among the most preferred and prudent choices [12]. These languages simplify the programming process by obviating the need to manage many low-level details of program execution (e.g., memory management), affording the programmer the ability to focus foremost on application logic, and to rapidly prototype programs in an interpreted and easily extensible environment. Any effort to choose from among these capable languages is ultimately founded in personal preference. Nonetheless, it should be noted that Perl and Python benefit from a relatively longer established tradition, and subsequently more widespread use in the field of bioinformatics. These facts should not serve to discourage the use of programming languages other than Perl or Python. Java, for example, which is popular in both academic curriculum and industry, has served as the basis for many successful bioinformatics projects. Nonetheless, programmers stand to benefit greatly from the many software tools, libraries, and educational materials available supporting the use of Perl and Python for bioinformatics [13]–[17].

In many cases, modern scripting languages can be “bridged” to other languages such that one is able to leverage the advanced features of other languages without abandoning the scripting language environment. Examples include the RPy library [18], which provides an interface between Python and the R language, and JRuby [19], a Java-based Ruby interpreter that enables interaction between the Ruby language and Java. Even if no formal scripting language interface is available for a particular software library, it is often possible to generate scripting language interface using tools such as the Simplified Wrapper and Interface Generator (SWIG) [20] or to simply “wrap” an existing executable using scripting language code. Through this paradigm, one becomes capable of envisioning composite solutions that incorporate the strengths of multiple language technologies, instead of being limited by the capabilities of a particular language.

Outside of programming languages there exists a multitude of software tools, libraries, and applications pertinent to various aspects of bioinformatics, and it is worthwhile to invest time in gaining broad knowledge of the most popular of such resources across the broad spectrum of bioinformatics. Additionally, we encourage proficiency in the use and maintenance of a Web server system, such as Apache [21], as a survey of the bioinformatics literature clearly demonstrates an increasing trend towards the Web-based development, delivery, and utilization of bioinformatics tools and services.

## The Benefits and Opportunities of Open Source Communities

Too often there is an urge among programmers to reinvent the wheel despite the availability of existing solutions. In some cases this can be an innocent and useful learning exercise, yet in most cases, this is an improvident and wasteful exercise. For many common problems in bioinformatics (e.g., parsing file formats or working with nucleotide data), it is often the case that others have previously implemented a solution to the problem, and in many cases these solutions are easily found implemented in open source software in the public domain. While general Internet search engines can be useful in locating existing bioinformatics source code, there are specialized search engines, such as Koders [22] and Google Code Search [23], that are specially designed to search across the public domain source code. These specialized search engines offer code-specific search options, such as the ability to constrain the search to specific programming languages or software licensing schemes. It is worthwhile to use these tools to search the public domain for existing open source code that might serve as inspiration for your own program code, or even repurposed as the basis for your own projects. It should be noted, however, that if the decision to repurpose open source code is made, it is recommended to fully understand the nature of the license under which open source code is distributed and to ensure that the redistribution terms set forth by the original authors are respected. Furthermore, as the modern bioinformatician will invariably benefit from the vast body of open source code in the public domain, it is good citizenship to contribute your bioinformatics source code into the public domain under an open source license when possible.

In bioinformatics, it is fortunate that solutions to many common tasks and problems have been codified into standardized, open source software frameworks [24]. These frameworks are often comprehensive, rigorously tested, documented, and engaged by vibrant and helpful user communities. Language-specific, open source bioinformatics frameworks are at the forefront of this effort, with BioPERL [25],[26], BioPython [27], BioRuby [28],[29], BioJava [30], and BioConductor [31] emerging as some of the most mature and widely used frameworks. Outside of pure bioinformatics there are a number of useful open source frameworks worth investigating, such as the SciPy [32] and NumPy [33] for scientific computing in Python and Ruby on Rails [34] for rapid Web application development. We would also urge those newer to bioinformatics and programming in general to engage these software framework communities as both a user and a contributor. Taking the time to understand the source code behind these frameworks and their system design can be highly educational, and members of framework user communities are often more than willing to constructively critique another's source code and program designs. Furthermore, active participation in an open source bioinformatics project can be noted on one's resume or CV as “on the job” bioinformatics experience, which can often be hard to gain for fledgling students and practitioners of bioinformatics.

## The Importance of UNIX Skills

Even if you don't choose to run a UNIX-based Operating System (OS) on your personal workstation, knowledge of UNIX is tremendously useful in bioinformatics. Although the Windows platform is perfectly adequate for bioinformatics, the simple truth is that the majority of bioinformatics computation happens on UNIX-based computer systems. A portion of this circumstance may be attributable to a tradition of scientific computing on UNIX and the availability of many free, open source UNIX-based OS, such as Linux. Even so, it can be argued that a UNIX-based OS offers several advantages when it comes to facilitating bioinformatics. Perhaps one of the most compelling reasons to learn UNIX is to avoid programming altogether by leveraging the flexible and extensible UNIX shell environment. UNIX systems provide access to a vast array of specialized utilities that are executed by a command interpreter known as the UNIX shell. While these commands are often limited to very specialized functionality (e.g., the “cat” command simply concatenates and prints files), the UNIX pipe operator, “|”, makes it possible to create ad hoc software pipelines by connecting the output of one command to the input of another. The software pipeline paradigm is common in bioinformatics [35], where many biological questions are evaluated by chaining specialized bioinformatics tools together into an analysis pipeline (e.g., BLAST search → Multiple sequence alignment→ Phylogenetic analysis) using a scripting language. In many cases, it is possible to avoid time-consuming and mundane programming tasks by simply chaining together a number of UNIX commands using the pipe operator (e.g., cut -f1 results.txt | grep “miRNA” | sed s/T/U/ > outfile.txt). It is also trivial to execute these utilities from within a program script to provide discrete functionality in place of additional script code (e.g., invoking the “gzip” utility to compress data files).

In the past, access to UNIX-based systems was fairly limited, and programmers typically gained text-based terminal access to UNIX-based systems by logging in to expensive, proprietary computer systems housed in university computing labs and research centers. Today it is possible to install a variety of user-friendly UNIX-based systems, such as Mac OS X or the open source Ubuntu Linux distribution [36], on a personal computer. There are even specialized Linux distributions available, such as BioBrew [37], which have been specially designed to support bioinformatics computing. ortunately the Cygwin project [38] brings a large degree of UNIX functionality to Windows-based systems; nonetheless there exist many bioinformatics tools and libraries that run only on, or are optimized specifically for, UNIX-based systems.

## Keeping Projects Documented and Manageable

It's difficult to produce clean, error-free, and reusable code without good programming hygiene. This includes using a clear and consistent variable naming convention, documenting your code, and for sufficiently large and complex projects, testing and building your code on a regular basis. Unfortunately, much like the many tasks associated with physical hygiene, these activities are often tedious, mundane, and apt to spark bemoaning or outright disregard by those expected to participate in them. Nonetheless the world of computer programming is fortunate to have a wealth of tools available whose sole purpose is to automate many of its mundane aspects.

In this era of open source software and collaborative research, it's likely that the program code you write will provide value to others. In fact, many journals will now require you to publish your source code along with a manuscript. Good documentation is key to making good sense of code, but it is often neglected due to its tedious nature, or the oft-erroneous belief that neither yourself nor anyone else will ever use the source code again. The best way to get into the habit of good code documentation is to automate it. Tools like Doxygen [39], JavaDoc [40], PyDoc [41], and others offer lightweight means for documenting code and easily generating formalized code documentation. Good variable naming is also an important aspect of good documentation. Resist the urge to use non-descriptive names for your variables (e.g., *a1* or *var1*) and try to be as consistent and verbose as necessary (e.g., *database_connection*). Many programming language communities have established preferred variable naming conventions [42], and therefore it is advisable to see if such conventions exist for the languages you use regularly. Well-documented code is easy for others to use, and if people can easily use your code, it's likely that the value you are providing to others will translate into increased opportunities personally and professionally. Also, many potential employers will want to see code you've written, and therefore it's beneficial to have a large portfolio of well-documented program code on hand for job interviews.

If you are working on a sufficiently large and complex bioinformatics project, it's likely that you may need to regularly build and test your software, deploy it to various Web server systems, or execute complex computational tasks as part of the work. Fortunately this process can be extensively automated by a number of freely available task automation software tools. The venerable UNIX “make” utility [43] is somewhat of a progenitor of many modern task automation tools. At its core, “make” will read and execute any number of tasks from a Makefile, which are defined using a structured macro language. Modern variants of “make,” such as Apache Ant [44], SCons [45], and Rake [46], offer functionality similar to “make” and are sometimes more intuitive to work with. Given their generalized nature and extensibility, programmers will find that nearly every aspect of building, testing, packaging, deploying, and executing program code can be automated using some form of task automation software.

## Preserving Your Source Code

Perhaps the only certainties in computer programming are that (i) there is a high probability that you will introduce new bugs every time you modify your code and (ii) your computer hardware accumulates an increased prior probability of failure over its lifetime. Despite this, many programmers are content to keep their precious source code strewn across their disk drives in the form of disordered, non-redundant files. Several Version Control Systems (VCS), which keep track of changes to source code files over time and offer the ability to revert and merge changes, are freely available. Despite the benefits offered by VCS, these systems remain underutilized by many programmers, and particularly in academic settings. Open source VCS such as CVS [47], Subversion [48], and Git [49] are simple to obtain, set up, and use, and many easy-to-use front end clients for these systems are freely available. The majority of modern text and source code editors also have support for VCS built in or offered through a plug-in or extension. VCS clients such as TortiseSVN [50] and SCPlugin [51] can even integrate VCS functionality at the OS file system level, such that source code versioning functionality is available through the OS file explorer utilities. Given their ease of use and low barrier of entry, there is almost no excuse for managing your source code outside of a VCS. If you are working on source code as a team, then use of VCS is a necessity, as they offer features such as file locking and automated change merging in cases where multiple people are modifying the same source code files. It is not necessary for one to set up and maintain their own VCS server system, as many free online services, such as SourceForge [52] and GitHub [53], offer standard VCS capabilities with many added features. The use of VCS can also be expanded beyond source code and is often used by academics to track and manage multiple versions of grants and manuscripts. Furthermore, many jobs in academia and especially industry will require the use of a VCS. Therefore experience with such systems will serve to enhance a personal and professional career in bioinformatics.

Many programmers also fail to realize the importance of backing up their computer systems until they've suffered a loss of their valuable source code through hardware failure, theft, or otherwise. Historically, computer backups required the involvement of IT departments, expensive backup software systems, and explicit scheduling of backup events. It is likely that these factors have contributed to the underutilization of backup software among students and cost-conscious academics. Recently, a new model of continuous, incremental computer backups, sometimes referred to as “snapshotting,” has emerged from a number of vendors and Web application service providers. These services, such as Mozy [54] and IDrive [55] (commercial services are given for example only), install a software client on a computer that monitors the computer for file system changes, streaming continuous backups of the computer's file system to encrypted, redundant online backup storage servers. The main advantage of these services is that after the initial setup, the software will continue to back up your files without any explicit intervention from the user (which is why they are sometimes also referred to as “set it and forget it” backup software). While most of these services are commercial endeavors, many offer free accounts that provide ample storage for source code and other important documents. At the time of this writing, open source implementations of such systems, such as TimeVault [56], are just beginning to emerge, but we expect many similar open source projects to appear and mature in the near future. Of course, backup software and systems themselves can fail; therefore it is provident to mitigate risk by implementing a redundant backup plan that incorporates two or more systems or services (e.g., backing up to an external hard disk and to an online backup service).

## Embracing Parallel Computing Paradigms

Parallel programming and execution can drastically enhance the speed of many computational tasks in bioinformatics, however the perceived complexity of parallel programming often serves to deter many from using it effectively in their bioinformatics work. There are essentially two major types of computational tasks that can be parallelized in software, which are defined by their dependency model as either Loosely Coupled (LC) or Tightly Coupled (TC). LC tasks are those whose execution does not depend on the state or output of any other computational task of the same class. Examples in bioinformatics would include a program that computes ligand-receptor binding affinities across many possible independent ligand-receptor combinations or a program that computes multiple sequence alignments for many independent protein families. LC tasks are generally the easiest to parallelize, as they often entail executing the same program logic on different data files or on the same data files using different parameters. There are many software systems available that are designed to facilitate the execution and control of LC task parallelization. Among the most popular are open source systems such as Sun Grid Engine (SGE) [57] and Open Portable Batch System (OpenPBS) [58]. Such systems are often referred to as job scheduling or batch processing systems, and they are routinely used to distribute individual computational tasks across groups of networked computers.

TC tasks are those whose execution is dependent on the state or output of other tasks. Examples in bioinformatics include molecular dynamics simulations or stochastic optimization heuristics. TC tasks are generally more difficult to implement, as they typically require programs to incorporate calls to functions from parallelization libraries, such as Message Passing Interface (MPI)–based libraries [59], and leave many complex details of parallel execution, synchronization, and consistency checking to the programmer.

Recently, a new paradigm for parallel computing, commonly referred to as MapReduce [60], was introduced by Google as a simplified software framework for parallelizing computation across large clusters of commodity computers. Since it was initially described, a large number of open source MapReduce projects have been implemented in various programming languages, such as Hadoop (Java) [61], Disco (Python) [62], and Skynet (Ruby) [63]. In essence, MapReduce frameworks help to break tasks down into discrete sub-problems (the *Map* step), which are distributed to networked compute nodes, and cohesively aggregate the results of the independent sub-tasks (the *Reduce* step). Although MapReduce is not suitable in every case where parallelization may be needed, many bioinformaticians are experimenting with MapReduce [64], and it is already showing great promise in accelerating short read mapping from high-throughput sequencing data sets [65].

It is important to note that it is not necessary to have access to a formal computing cluster to utilize parallel computation. Most of the software frameworks that facilitate parallel computing can execute parallel processes across multiple CPUs on a single machine. At the time of this writing, a computer workstation with 8 CPU cores can be purchased for less than US$3,000.00; thus substantial parallel computing capabilities can be rather easily obtained by even those with the most modest budgets. Furthermore, virtualized, or “cloud,” computing services, such as the Amazon Elastic Compute Cloud (EC2) [66], provide an economical means to procure vast computing resources to facilitate parallel computation on an as-needed basis. Consequently, large, publicly funded biocomputing initiatives, such as the Cancer Biomedical Informatics Grid (caBIG) [67], have begun to investigate such cloud computing architectures to support their efforts.

## Structuring Data for Speed and Scalability

The tradition of using flat files in bioinformatics (i.e., storing data records in large text files) is out of step with current needs. In the modern era of integrative biology and medicine, we are often faced with the task of integrating data from multiple sources in complex ways (e.g., relating SNPs, gene expression, and proteomics data to build models of gene regulation). The use of flat files often requires the programmer to load huge numbers of data records into system memory, and then index and join these data using custom program logic. Relational Database Management Systems (RDBMS), such as MySQL [68], are well suited for such tasks, yet they remain underutilized by many in bioinformatics. The utilization of RDBMS can be intimidating to those without formal database training, as they often require the set-up and management of database server systems, and their contents must be defined and queried using the somewhat peculiar Structured Query Language (SQL).

The conceptual incongruities between RDBMS and modern object-oriented programming paradigms have spurred the development of Object Relational Mapping (ORM) frameworks, which provide language-specific, object-oriented interfaces to traditional RDBMS. ORMs virtually eliminate the need to write SQL statements to interact with the RDBMS. Instead, a programmer instantiates native language data structures (typically an object sub-classed from an ancestor class defined by the ORM framework), and calls to methods of these data structures are automatically translated by the ORM into the appropriate SQL query statements. ORMs work bidirectionally, such that any results returned by the database are also translated into native language data structures. For a simple example, an invocation of the following ORM pseudo code:

translated_sequence = ProteinSequence. find(10)

might automatically generate the following SQL statement:

SELECT * FROM protein_sequences WHERE id = 10”

The ORM would then automatically execute the statement in the RDBMS and use the results of the query to instantiate the variable translated_sequence as an object of the class ProteinSequence whose attributes and data match those defined by the fields in the translated_sequence table row with the key field id = 10. A potential downside of ORMs is that many require the database structure to conform to a predefined convention, making it sometimes difficult to use ORMs with existing databases. Also, the SQL queries generated by ORMs can sometimes make suboptimal use of the database's indexing and joining capabilities. Popular open source ORMs include ActiveRecord (Ruby) [69], SQLObject (Python) [70], Hibernate (Java) [70], and DBIx::Class (Perl) [71].

There are a number of alternative database systems that offer many of the advantages of RDBMS without the overhead of server set-up and maintenance. SQLite is a fully embeddable, server-less RDBMS engine that allows for the creation of portable, relational database files that can be queried using SQL via a lightweight C library, for which many high-level and scripting language interfaces are available. SQLite can also be used in conjunction with many ORM frameworks, drastically reducing the complexity of incorporating fast, structured data storage into bioinformatics scripts and applications. Another server-less database system is BerkeleyDB, which also integrates into software via a lightweight C library but differs in that it offers a simpler key/data model rather than a relational data model. For many bioinformatics tasks, we seek to integrate data objects by unique identifiers (e.g., matching gene expression and SNP data by Entrez GeneID), which is particularly amenable to the key/data paradigm behind hash-like database systems such as BerkeleyDB.

The key/data database model has proven to be particularly scalable, forming the conceptual basis of a new breed of distributed, large-scale database systems used to crawl Internet-scale (i.e., multi-terabyte) datasets. Open source implementations of these systems include HBase [72], Hypertable [73], and Cassandra [74], which are being used by some of the world's largest Internet companies, often in conjunction with MapReduce-based parallel computations. These database systems are also well suited for working with bioinformatics data of similar scale. Also worth investigating are so-called “schema-less” or “document-oriented” database systems, in which database objects can be defined in an ad hoc manner using key/data field definitions. Examples include CouchDB [75], MongoDB [76], and Tokyo Cabinet [77]. These systems offer more flexible query interfaces with optimizations for Web-based applications and are already showing some promise in the development of bioinformatics Web applications [78].

## Understand the Capabilities of Hardware

Although we advise the use of high-level scripting languages for many aspects of bioinformatics, it is still important to understand how various features of modern computer hardware architectures can be leveraged to substantially enhance and accelerate bioinformatics. Many recent innovations in computer hardware designs were born from the needs of 3D computer gaming, where the mathematical and computational needs are oftentimes on par with that of bioinformatics. Therefore bioinformaticians can and have repurposed these technologies to enhance and accelerate a broad range of tasks in bioinformatics, and in many cases, to dramatic effect [79].

One straightforward means of using hardware to accelerate bioinformatics code is to vectorize its execution using the Single Instruction, Multiple Data (SIMD) instruction sets offered by all modern workstation CPUs. SIMD capabilities are referred to by different names depending on implementation and manufacturer (e.g., SSE in Intel x86 and Altivec in PowerPC CPUs), but their overall purpose and capabilities are essentially the same. The extent of code vectorization possible using SIMD is dependent on various features of the CPU vector units, but generally, SIMD allows a set of instructions that would normally be executed serially (e.g., a “for” loop of 1..*n* floating point calculation) to be executed in parallel per CPU cycle (e.g., four loop iterations at a time). Many free and commercial compilers now have auto-vectorization capabilities, which attempt to analyze your code and automatically optimize sections of program execution using SIMD when possible. Therefore existing bioinformatics applications may find speed gains through a simple recompiling of the source code with the compiler's SIMD optimization capabilities enabled. SIMD optimization has already been used in bioinformatics to substantially improve the performance of many sequence matching and alignment algorithms [80]–[83].

The vector computing paradigms ushered in by SIMD have been extended towards the development of specialized Graphics Processing Units (GPU), which act independently from the primary CPU(s) to process 2D and 3D graphics rendering. Because of their computational prowess for certain types of mathematical computations and transformations, which can be up to several orders of magnitude faster than the primary CPU(s) for similar tasks, GPUs have been appropriated for tasks beyond graphics processing, engendering the development of several techniques for General Purpose computing on GPU (GPGPU). GPGPU is facilitated by a number of software frameworks, such as CUDA [84] and OpenCL [85], which aim to provide generalized programming interfaces to the GPU hardware. Not surprisingly, GPGPU has already been successfully harnessed by bioinformaticians to drastically accelerate tasks related to sequence alignment [86],[87] and molecular dynamics simulations [88]. Interestingly, a number of scripting language interfaces are being developed for GPGPU libraries, such as gputools (R) [89] and pystream (Python) [90], making GPGPU hardware acceleration capabilities accessible to those who are most comfortable working with high-level languages.

Another hardware technology of note for bioinformatics is the Field Programmable Gate Array (FPGA). An FPGA can be loosely conceptualized as a dynamically reconfigurable CPU, where the logical elements found within the chip can be dynamically reconfigured using a specialized hardware description language. The benefit of FPGAs for bioinformatics comes from the fact that it is possible to implement certain types of bioinformatics algorithms within the FPGA, effectively enabling the creation of customized hardware acceleration for bioinformatics computations. FPGA-based hardware acceleration has already been demonstrated for several bioinformatics applications, including sequence alignment [91]–[93], molecular dynamics [94], and proteomics [95]. Additionally, a number of specialized FPGAs for bioinformatics applications can be readily purchased “off the shelf” from commercial vendors.

## Embracing Standards and Interoperability

Data exchange and interoperability is an old problem in bioinformatics that has engendered the development of a number of standardized data file formats. However, efforts to standardize data are often contentious and slow to keep pace with emerging data types. While it is most noble to use an established, standardized data format when possible, it is sometimes not possible or practical. Still, there are habits that can be put into practice that make it easier to share your data with others. The most basic approach is to use a markup language, such as the eXtensible Markup Language (XML), to provide some basic structure and annotation for your data format. XML parsers or XML Stylesheet Language Transformations (XSLT) can be easily used to convert data structured by XML to any number of alternative formats. Although XML may be seen as overkill for simpler data formats, efforts should still be made to provide your data in a format that is easily consumable by others. Comma- or tab-delimited file formats are a commonplace means for representing data when data can be represented in tabular form, however this approach is not practical when the format is required to define complex relationships between entities, nor do they permit encapsulated nesting of data elements within others. Although XML would be applicable in such situations, its use can be cumbersome to those unfamiliar with it; therefore one could alternatively use the lightweight data serialization formats such as JavaScript Object Notation (JSON) [96] or YAML [97] to provide a lightweight data format that is easily interpreted by both humans and programming languages.

Another alternative when it comes to facilitating data exchange is to avoid file formats altogether, and instead provide programmatically accessible, Web-based interfaces to your data sources. One means to do this is to expose your data sources using standardized Web-service interfaces, such as Representational State Transfer (ReST) [98] or Web Services Description Language (WSDL) [99], for which many counterpart implementation libraries exist for a large number of programming languages. This would allow others to access your data sources directly from program code, potentially reducing the burden for sharing large data sets with collaborators and communities.

When providing data for others, it is also important to use as many standardized data and concept identifiers as possible. It is unfortunate that so much effort has gone into the development of text mining tools and techniques for identifying which species, diseases, and drugs are represented in public datasets, because many of these concepts are defined by these data using inconsistent, free-text labels. If the designers of these file formats and data repositories had required the use of the many systematized nomenclatures available, such as the many defined within the freely available Unified Medical Language System (UMLS) [100], then it would be much easier to systematically query and appropriate these vast public data repositories for downstream research. Online services such as The Open Biomedical Ontologies Foundry [101] and the NCBO BioPortal [102] offer rich, Web-based interfaces for discovering and exploring a large number of existing biomedical ontologies available in the public domain.

## Value Your Time

The advent of open source software and the commoditization and virtualization of computing hardware have drastically reduced the cost of software development. By far, the most expensive aspect of software development today is the programmer's time, and thus the success of programming efforts in academia or industry will be invariably tied to effective use of programmer time. One major source of time inefficiency in software development is an imbalance of architecture versus accomplishment. The urge to create this imbalance is particularly strong when developing large, object-oriented systems, where programmers might be inclined to code excessively complex data models, in an effort to build a system that accounts for all possible points of failure and edge cases. There is always room to improve a system to make it more “perfect.” Therefore in regards to program design, we assert it is best to invoke Voltaire's adage, “The perfect is the enemy of the good.” The most highly used and cited bioinformatics tools simply work well enough to do a reasonably good job at the specific task for which they were designed. The success of bioinformatics software is based not on the elegance of the software design, but rather its utility as a tool for driving and answering biological questions. Consequently it is no surprise that many successful bioinformatics apps are written by biologists who lack formal computer science training, as they undoubtedly put scientific utility ahead of architectural elegance and completeness.

The key to effective use of programming time is to put a high value on your time. As a guide, it can be helpful to put a value on your time based on your salary, stipend, or personal goals. If you determine your programming time to be worth $100 an hour, is it reasonable to take the time to re-implement a statistical method in code if you can purchase a commercial software library that can provide it for $50? Is it reasonable to spend weeks to optimize an algorithm if $2,000 in additional computing hardware will accomplish the same performance gains? As a general principle, outsource or purchase everything but genius to maximize your contribution to driving scientific questions and accomplishment.

## Conclusion

Although there are many factors and principals underlying excellence in bioinformatics, the rules presented here aim to convey a set of pragmatic knowledge and principals that are most likely to offer high value to programmers across the broad spectrum of bioinformatics in both academia and industry. The relevance of many of the rules outlined here can be directly evaluated though a survey of the bioinformatics positions described within scientific job sites, such as Nature Jobs (http://www.naturejobs.com) and Science Jobs (http://www.sciencejobs.com). For example, at the time of this writing, a search for the term “UNIX” finds more than 100 open positions seeking proficiency in UNIX.

Readers should take note that the landscape of tools and technologies used in bioinformatics is constantly changing and that long-term success in bioinformatics requires one to stay abreast of these changes. Readers are encouraged to make use of newsreaders to subscribe to the RSS feeds of the many journals, blogs, and user community sites oriented towards bioinformatics. Readers are also encouraged to join the many vibrant bioinformatics user communities established within popular social networking sites, such as LinkedIn (http://www.linkedin.com), FriendFeed (http://www.friendfeed.com), Epernicus (http://www.epernicus.com), and Twine (http://www.twine.com).
